# Peer Review of “The Influence of SARS-CoV-2 Variants on National Case-Fatality Rates: Correlation and Validation Study”

**DOI:** 10.2196/38547

**Published:** 2022-05-24

**Authors:** Mathew Mbwogge

**Keywords:** SARS-CoV-2, COVID-19, variants of concern, case-fatality rates, virulence, vaccine effectiveness, correlation study

*This is a peer-review report submitted for the paper “The Influence of SARS-CoV-2 Variants on National Case-Fatality Rates: Correlation and Validation Study”*

## Round 1 Review

### General Comments

Emerging variants of concern (VOCs) have increased the uncertainty about bringing the pandemic to an end [1]. Countries will not only have to focus on stepping up vaccination efforts but effective surveillance as well to monitor and characterize the more transmissible and deadly variants [2-5]. The most prominent confirmed cases include Alpha, Delta, Beta, Eta, and Kappa [6]. This, in addition to flagging the need for more sustainable measures, raises concerns over their impact on case-fatality rates (CFRs) in different countries.

The authors of the paper [7] “The influence of SARS-CoV-2 variants on national case fatality rates” attempted to investigate the impact of VOCs on (1) proxy CFRs and (2) the vulnerability of persons living with comorbidities, using open source data of reported daily cases. They found little variations in the association between World Health Organization data-driven factors and the average proxy CFR and concluded that the increase in the impact of VOCs may be attributed to the fact that those living with comorbidities are more susceptible to infection severity. Other studies that evaluated the impact of new variants found them to be associated with higher rates of hospitalization and death. In the United Kingdom for instance, studies among cohorts infected with the B.1.1.7 variant (VOC-202012/1) compared to those with normal infections found an increased risk of hospitalization [4] and deaths [5,8,9] in the intervention group, using the TaqPath assay. According to expert opinion on some of these results, patients with the Kent or Delta variant (B.1.1.7) were 64% more likely to die [10]. The CFR was higher among men than women and increased with age.

This paper has been structured in compliance with the IMRD approach. The authors capitalized on prior published data and the concept on which the analysis was based [11] to generate new data, which seems logical. The English used is simple enough for the readership but demands improvement.

Even though the paper’s methods and analysis are based on a published concept, the fact that this was done by the same authors and no other authors have been cited making use of the same concept makes the paper’s methods weak. The study rationale has not been well established, thereby making the study objectives and research questions less robust. Besides, not only is data about variants of concern lacking and the interpretation of the results not well articulated, but the conclusion also arrived at is not clear enough in relation to the defined objectives. Kindly refer to the following major and minor comments.

### Specific Comments

#### Major Comments

1. Kindly refer to the journal guidelines to see how titles are formatted. Well-formatted titles should include the main outcome of interest, the subject matter, and the study design.

2. Your interest is to measure the influence of VOCs, not SARS-CoV-2 variants as reflected in your title. You may want to correct that.

3. Your abstract must include (1) Background, (2) Objective, (3) Methods, (4) Results, and (5) Conclusions. Kindly use this source to see how to structure your paper [12].

4. The phrase I quote “may increase the vulnerability of persons with certain comorbidities” in the Abstract is not an objective. Kindly rephrase together with the first objective that appears too long.

5. You need to include (1) Study Rationale and (2) Specific Objectives in your Introduction as subsections. The “Specific Objectives” subsection should normally be the last part of your Introduction.

6. In your Study Rationale, make efforts to trace other studies that have made use of similar methods in predicting the impact of VOCs. This section needs to at least include some basic data about VOCs (prevalence or impact on hospitalizations and mortality). You may want to make use of this reference [6].

7. Given that this paper is based on VOCs, it would be sensible to include in your Introduction and as part of your background literature evidence of a literature review of the different VOCs (their characteristics and virulence). Readers will be keen to discover the new variants in circulation. The availability of data on VOCs and variants under investigation is key because it flags the need for vaccination, increases uptake, and signals policy makers about the importance of modifying surveillance policies.

8. If you decide to include research questions or hypotheses to be tested in your paper, kindly associate these with your research objectives. This makes it easy for readers to see how you transformed each objective into a question, as well as the hypothesis to be tested.

9. Kindly start your Methods section with the subsection “Study Design” and clearly state your study design. This is particularly important not just for reviewers but for those undertaking systematic reviews.

Studies are often excluded or not simply traced as a result of a lack of a clearly stated research design. Besides, it is the place of the author to inform readers of the study design and not for readers to determine the design that was used. Authors making use of study designs that are new to the journal’s readership always make an effort to cite articles making use of similar designs regarding the subject matter.

10. I suggest structuring your Methods section as follows:

- Study design
- Data sources and setting (including providing a brief description of each country being profiled and the triggers and specific reasons for choosing particular countries to include in your analysis)
- Study variables/outcomes (kindly specify here, the comorbidities you were interested in together with definitions for outcomes like case fatality)
- Data analysis (include equations here and specify any underlying assumptions). Clearly explain how you run the correlations and time series, and report any statistical program that was used.

11. Explain how adjustments for age, sex, ethnicity, type of VOC, seasonality, etc, in the correlations were made. For instance, the impact on the national CFR may be contingent on the type of variant [13]. Comorbidities may exacerbate during winter and make it difficult to attribute increased mortality among those with comorbidities to VOCs [10].

12. In your data analysis, kindly explain how you arrived at using the Pearson product moment correlation. Kindly justify if your data was linear and report the values of normality tests that were performed prior to choosing the approach of analysis.

13. Kindly report how the different linearity assumptions were verified (for linear data).

14. In your data analysis, kindly report how you determined the strength of association between the proxy national CFRs and the different covariates.

15. The Results section seems to be a mix of data analysis, results, and discussion. Kindly move texts relating to the above to their respective subsections. For instance, readers will not expect to see any explanations in the Results section as this should normally appear under discussion, where you normally should explain why results appear the way they are. Additionally, equations relating to data analysis should not appear under results.

16. A look at your study results shows that this paper has 3 objectives I state (1) to assess the fluctuations in the daily proxy national CFRs, (2) to investigate the correlation between average national proxy CFRs and potential cofactors/comorbidities, and (3) to describe the correlation between proxy national CFRs of country pairs by region. You might want to amend your study objectives accordingly.

17. I suggest you organize and report your results by objective (1, 2, and 3) for a better flow.

18. You reported to have made use of the Pearson correlation coefficient but have not reported the coefficients obtained from the correlation anywhere. Kindly clarify.

19. Kindly structure the Discussion section following the journal guidelines. I suggest:

- Summary Findings
- Strength and Limitations
- Interpretation of Results
  - Fluctuations in the daily proxy national CFRs
  - Linear correlation of the averaged CFR and potential cofactors
  - Linear correlation between proxy CFRs for country pairs by region
- Implications for Policy and Research
- Conclusion

20. Your need to compare your results with those of other studies in your “Interpretation of Results” in your discussion, by citing other studies on the same subject matter and preferably undertaken in the same countries being profiled. This helps to situate the study within the existing literature. I understand this might be challenging for some objectives. Kindly provide explanations for the results in the event of a lack of suitable studies.

21. Your conclusion needs to state your results within the context of your study objectives and give the significance and implications to future research, surveillance, and policy.

22. Kindly refer to the guidelines for referencing or have a look at published articles in the journal to which this work is submitted. Your references need to follow the AMA citation style. Please refer to the references of this report.

#### Minor Comments

23. The Methods subsection of your Abstract needs to summarize your study design, data sources, and how data was analyzed including any statistical packages.

24. Kindly ensure that the conclusion of your paper is under the subtitle “Conclusion.”

25. Move all abbreviations to the end or as the last section of your paper.

26. Please be aware that you are not allowed to include more than 8 figures in your paper. You may want to merge some and move others to multimedia appendices. I did not find Figure 2 very necessary and you might want to move that.

27. All figures to be published in the body of your paper must also be uploaded online. Kindly refer to the journal guidelines.

28. I suggest moving Table A to the “Data Sources and Setting” subsection and labeling it as Table 1.

29. You need to cite more papers including those from the journal to which you submitted.

30. Kindly include a PubMed ID at the end, for each reference (searchable at crossref.org). Kindly refer to the references in this peer-review report.

31. Endeavor to cite the PDF version of articles for all web links if possible.

#### Round 2 Review

##### General Comments

I am happy that the authors of the paper titled “SARS-CoV-2 variants of concern: Influences on national case fatality rates” have addressed all concerns raised in the previous round, thereby giving the paper a new and improved outlook. However, these have not been addressed in a manner satisfactory enough. The study title even though modified from “The influence of SARS-CoV-2 variants on national case fatality rates” still needs to comply with the journal guidelines [12]. The study objectives are not consistent across the different sections. Some sections need to be reorganized for a better flow. The English used for reporting warrants improvement. Kindly refer to the below minor comments to improve the paper further.

##### Specific comments

###### Minor comments

1. Could you please identify this study as a “Correlation Study” [13]? For instance “The influence of SARS-CoV-2 variants on national case-fatality rates: Correlation and Validation Study”

2. The current text in the Results subsection of the Abstract should be part of the Methods subsection of the Abstract. Kindly move it to the start of your Methods subsection. Could you please summarize your findings into say 5 to 10 lines in the Results section of your Abstract? One will expect to see some figures reported from the main results in this subsection. You may want to ensure that your word count for the Abstract is not above 450 by decreasing the word count in your Methods and Conclusions subsections.

3. The discoverability of your paper can be improved by including SARS-CoV-2, COVID-19, and 2019-nCoV in your keywords. Kindly modify “Country correlation” to “Correlation study.”

4. The Objectives section of your Introduction seems to include the study background information; otherwise, I do not understand why it should be that lengthy. Kindly move the subtitle “Objectives” (better phrased as “Specific Objectives”) to the end of your Introduction and state your specific objectives. The Objectives subsection should not be more than a paragraph. All other text should either be part of your study background literature or rationale. The Specific Objectives subsection should be formatted as follows:

“Specific Objectives

The principal objectives of this study are to (1) establish a valid proxy national CFR and assess its daily fluctuations, (2) investigate the correlation between average national proxy CFRs and potential cofactors/comorbidities on a global and regional basis, and (3) describe the correlation between proxy national CFRs of country pairs by region.”

Please do not include any other text before the Methods section. Additionally, kindly ensure that the above specific objectives and those in your Abstract are the same for consistency.

5. The use of the word “reference” in most of your statements (eg, “To evaluate any changes in the susceptibility to co-factors, one can follow the method introduced in reference”) may not be appropriate. I suggest you state author names instead of using “reference” when referring to a particular research work. Kindly rephrase these all through the body of the manuscript.

6. For standard reporting and to be in line with the journal guidelines, I suggest replacing the title “Method of Analysis” with “Methods.” It will be good to identify this study as a “Correlation and Validation” study under your “Study Design” subsection. This should be a single statement or at most 5 lines if you need to explain why you used the design and make reference to other papers.

7. Regarding your analysis approach in the study methods, it will be good to provide a few lines on how each of the assumptions for running a Pearson product moment correlation was satisfied [14].

8. Kindly change the title “Discussion and Conclusion” to “Discussion.” I still suggest you structure your Discussion in line with the journal guidelines [15]. You may want to refer to papers published in JMIR to help you with how to structure the Discussion section. Based on journal guidelines, well organized and standard Discussion sections will bring out the subtitles (not as paragraphs) “Summary of Findings,” Study Limitations,” “Comparison With Prior Studies,” and the “Conclusion.” Even in a situation where you do not have enough papers to cite under “Comparison With Prior Studies,” the subsection will still include your reasons and explanations of why results appear the way they do.

9. I guess your current Conclusion that appears quite lengthy includes materials for the Discussion section. Kindly size down and move a majority of the material to the Discussion section (specifically to the “Comparison With Prior Studies” subsection).

10. I note that the “Summary of Findings” in the Discussion should be a carbon print in terms of length and text of the “Results” subsection in the Abstract. For coherence and consistency, the more you can make these the same, the better. The same should be the case with the “Objectives” subsection in the Abstract and the “Specific Objectives” subsection at the end of your Introduction.

11. Kindly define a study aim in one sentence based on your 3 specific objectives and start your Conclusion with this study aim. This reminds readers of what you set out to do and helps them marry it with what you found. This should be followed by the main findings in just a few lines, lessons learned, what the findings mean for public health, and future research.

12. Just like the “Summary of findings,” it is common practice not to expect the Conclusion of a paper to be lengthy since all explanations relating to the results should be part of your “Comparison With Prior Studies” subsection in the Discussion.

13. As per the journal guidelines, kindly move your Abbreviations subsection to after the references.

14. Ensure you follow the journal guidelines to report your P values.

## Abbreviations

CFR: case-fatality rate
VOC: case-fatality rate
